# The chloride channel family gene *CLCd* negatively regulates pathogen-associated molecular pattern (PAMP)-triggered immunity in *Arabidopsis*

**DOI:** 10.1093/jxb/ert484

**Published:** 2014-01-21

**Authors:** Wei Guo, Zhangli Zuo, Xi Cheng, Juan Sun, Huali Li, Legong Li, Jin-Long Qiu

**Affiliations:** ^1^State Key Laboratory of Plant Genomics, Institute of Microbiology, Chinese Academy of Sciences, Beijing 100101, China; ^2^School of Life Sciences, Capital Normal University, Beijing 100048, China

**Keywords:** *Arabidopsis thaliana*, *AtCLCd*, chloride channel, PAMP-triggered immunity.

## Abstract

Chloride channel (*CLC*) family genes are ubiquitous from prokaryotes to eukaryotes and encode proteins with both channel and transporter activities. The *Arabidopsis thaliana* genome encodes seven *CLC* genes, and their products are found in a variety of cellular compartments and have various physiological functions. However, a role for AtCLCs in plant innate immunity has not previously been demonstrated. Here it is reported that AtCLCd is a negative regulator of pathogen-associated molecular pattern (PAMP)-triggered immunity (PTI). T-DNA insertion mutants of *AtCLCd* exhibited enhanced responses to the elicitor, flg22. The PTI phenotypes of the *clcd* mutants were rescued by expression of *AtCLCd*. Overexpression of *AtCLCd* led to impaired flg22-induced responses. In line with a role for AtCLCd in PTI, the *clcd* mutants were more resistant to a virulent strain of the bacterial pathogen *Pseudomonas syringae* pv. *tomato* DC3000 when spray inoculated, while *AtCLCd*-overexpressing lines displayed increased susceptibility to this pathogen. Interestingly, flg22 treatment was found to repress the expression of *AtCLCd*. In addition, its expression was elevated in mutants of the flg22 pattern recognition receptor (PRR) FLS2 and the PRR regulatory proteins BAK1 and BKK1, and reduced in an *FLS2*-overexpressing line. These latter findings indicate that FLS2 complexes regulate the expression of *AtCLCd*, further supporting a role for *AtCLCd* in PTI.

## Summary

It is shown that AtCLCd negatively regulates *Arabidopsis* PTI, probably by interacting with the PRR signalling pathway. Its sequence indicates that *AtCLCd* encodes a chloride/proton exchanger.

## Introduction

Under natural conditions, plants are constantly exposed to harmful pathogens. Plants, being sessile, cannot simply escape from biotic stresses. However, they have evolved a complicated innate immune system to fight pathogen attacks. Their innate immune system generally consists of two layers of defence (Jones and Dangl, 2006). The first, named PAMP-triggered immunity (PTI), is triggered by the recognition of pathogen-associated molecular patterns (PAMPs) by plant cell surface pattern recognition receptors (PRRs). Adapted pathogens can secrete effector proteins into host cells to suppress PTI. The second layer of defence, termed effector-triggered immunity (ETI), originates in the cytoplasm and is triggered directly or indirectly by the recognition of secreted microbial effectors by plant resistance (R) proteins. Thus, activation of plant innate immunity is largely dependent on recognition of ‘non-self’ signals (Robatzek and Saijo, 2008; Boller and He, 2009; Tsuda and Katagiri, 2010).

After perception of PAMPs or effectors, host cells initiate a series of physiological processes, of which the oxidative burst, extracellular alkalinization, and protein phosphorylation are the earliest (Felix *et al.*, 1999; Bauer *et al.*, 2001; Kunze *et al.*, 2004). Extracellular alkalization is caused by ion fluxes across the plasma membrane (Nürnberger *et al.*, 1994; Jabs *et al.*, 1997), indicating that ion channels are activated in response to pathogen attacks. A number of channels have been shown to play a role in plant defence responses (Jeworutzki *et al.*, 2010; Qi *et al.*, 2010; Koers *et al.*, 2011). Anion channels have been shown to be required, especially for PTI. Elicitors or PAMPs such as cryptogein and flagellin can induce massive anion efflux (Wendehenne *et al.*, 2002; Jeworutzki *et al.*, 2010). The production of reactive oxygen species (ROS) is a common early response to PAMPs. Using a pharmacological approach, Colcombet *et al.* (2009) showed that rapid-type (R-type) anion channels are important in flagellin-induced ROS production in *Arabidopsis* suspension cells, and Jeworutzki *et al.* (2010) recorded strong anion currents in mesophyll and root hair cells of *Arabidopsis* upon PAMP treatment. In addition, inhibition of anion channels impaired the cryptogein-induced cell death in the hypersensitive response (HR) of tobacco suspension cells (Wendehenne *et al.*, 2002). PAMP-triggered plasma membrane anion channel opening was found to be dependent on PRRs and BAK1, suggesting that the anion channels are activated downstream of PRRs (Jeworutzki *et al.*, 2010).

Several gene families encoding anion channels/transporters have been identified in plants. Of these, three families, *SLAC1* (slow anion channel 1), *ALMT1* (aluminium-activated malate transporter 1), and *CLC* (chloride channel), have been the most studied (Barbier-Brygoo *et al.*, 2011). Patch–clamp studies on *Vicia faba* guard cells revealed the presence of R-type and slow-type (S-type) anion channels (Schroeder and Keller, 1992). The S-type channel is encoded by *SLAC1* (Vahisalu *et al.*, 2008), and the R-type channel by members of the ALMT transporter family (Meyer *et al.*, 2010). Interestingly, barley powdery mildew was shown to activate host cell S-type anion channels and thereby inhibit light-induced stomatal opening (Koers *et al.*, 2011). Similarly, the *Arabidopsis* guard cell SLAC1 was found to be necessary for stomatal closure in response to biotic stress (Negi *et al.*, 2008; Saji *et al.*, 2008; Vahisalu *et al.*, 2008; Montillet *et al.*, 2013). Despite the evidence of the involvement of plant anion channels in defence responses, there is so far little direct evidence of the participation of anion channels in innate immunity, and how these channels regulate plant defence responses also remains elusive.

CLC family proteins are present in prokaryotes and eukaryotes and have both channel and transporter activities (Jentsch, 2008; Barbier-Brygoo *et al.*, 2011). The *Arabidopsis* genome encodes seven *AtCLC* genes (*AtCLCa–AtCLCg*) (Hechenberger *et al.*, 1996; Lv *et al.*, 2009), and their products are found in several cellular compartments including the vacuole membrane (AtCLCa, AtCLCb, and AtCLCc) (De Angeli *et al.*, 2007; von der Fecht-Bartenbach *et al.*, 2010), the Golgi apparatus (AtCLCd and AtCLCf), and chloroplast membranes (AtCLCe) (Marmagne *et al.*, 2007; von der Fecht-Bartenbach *et al.*, 2007). AtCLCa, AtCLCb, and AtCLCe are required to maintain normal cellular nitrate levels (De Angeli *et al.*, 2007; von der Fecht-Bartenbach *et al.*, 2010), and AtCLCc participates in both nitrate and chloride homeostasis and regulates stomatal movement and salt tolerance (Jossier *et al.*, 2010). AtCLCd has been proposed to regulate luminal pH in the *trans*-Golgi network (TGN) (von der Fecht-Bartenbach *et al.*, 2007). However, a role for AtCLCs in plant innate immunity has not previously been demonstrated. In this work, the defence phenotypes of all available *AtCLC* family mutants were examined and it was found that *AtCLCd* negatively regulates PTI.

## Materials and methods

### Plant materials and growth conditions

The T-DNA insertion mutants *clcd-1* (SALK_042895), *clcd-2* (SALK_052368C), *clca* (CS857712), *clcb* (SALK_027349C), *clcc* (SALK_115644C), *clce* (SALK_010237), and *clcg* (SALK_087699) were obtained from the *Arabidopsis* Biological Resource Center. Homozygous T-DNA insertion lines were screened by PCR and confirmed by reverse transcription–PCR (RT–PCR) using gene-specific primers (see Supplementary Table S1 available at *JXB* online). *fls2* (SALK_141277) was kindly provided by Dr Jianmin Zhou. *bak1-5*, *bkk1-1*, and *bak1-5/bkk1-1* were kindly provided by Dr Cyril Zipfel. *Arabidopsis thaliana* wild-type Columbia-0 (Col-0) and mutants were grown in a growth room with 8h light/16h darkness at 75% relative humidity and 22 °C.

### Gene constructs and plant transformation

To complement the *clcd* mutant, the 1.6kb promoter region of *AtCLCd* and the full-length open reading frame were separately PCR amplified from *Arabidopsis* Col-0 genomic DNA and cDNA, respectively, and the NOS terminator was then cloned into the binary vector pCB302 (Xiang *et al.*, 1999) to create construct *pAtCLCd*:*AtCLCd*. To overexpress *AtCLCd*, the full-length coding sequence was amplified and cloned downstream of the *Cauliflower mosaic virus* (CaMV) 35S promoter in the binary vector pCB302-3 (Xiang *et al.*, 1999) to obtain *p35S:AtCLCd*. To overexpress *FLS2*, the full-length coding sequence was amplified from cDNA and cloned into binary vector pCAMBIA1300 (Cambia) between the *Bam*HI and *Spe*I multiple cloning sites to form *p35S*:*FLS2*. The primers used for making the constructs are listed in Supplementary Table S1 available at *JXB* online. All constructs were verified by sequencing and introduced into *Agrobacterium tumefaciens* strain GV3101 by electroporation. *Agrobacterium tumefaciens* carrying the constructs was used to transform *Arabidopsis* Col-0 by floral dip (Clough and Bent, 1998). Transformed *Arabidopsis* lines were selected on soil by spraying with a 1:1000 dilution of Basta (Bayer CropScience) or on MS (Murashige and Skoog) agar plates supplied with 20mg l^–1^ hygromycin B.

### Semi-quantitative and quantitative real-time RT–PCR

Total RNA was extracted from *Arabidopsis* leaves with TRIzol reagent (Invitrogen). A sample containing 2 μg of total RNA was treated with DNase I (Invitrogen) and reverse transcribed with M-MLV Reverse Transcriptase (Promega). For semi-quantitative PCR, 25 μl reaction mixtures contained 0.5U of *Taq* DNA polymerase (MBI, Fermentas), 80ng of cDNA, 200 μM of each dNTP, and 0.2 μM of each primer. PCR parameters were: 3min at 95 °C followed by 28 cycles of 95°C for 30 s, 60 °C for 30 s, and 72 °C for 40 s.

Real-time PCR was performed using TakaRa SYBR Premix Ex Taq following the manufacturer’s instructions, and run in a Bio-Rad CFX96 (Bio-Rad Laboratories). The reaction volume was 20 μl containing 10 μl of SYBR pre-mix, 0.5 μM of each primer, and 20ng of cDNA. A three-step protocol was used: a denaturation program (95 °C for 30 s), an amplification and quantification program repeated 40 times (95 °C for 5 s, 60 °C for 30 s and 72°C for 30 s with the fluorescence measurement), and a melting curve program (55–95 °C, with a 0.5 °C increment each cycle). Each sample was replicated three times. *ACTIN2* was used as an internal reference gene, and normalized fold expression was calculated employing CFX Manager Software (Bio-Rad) and the –ΔΔC(t) method. Unless otherwise indicated, result values displayed are relative to wild-type (Col-0) untreated plants, which are set to a relative value of 1. The primers used in semi-quantitative and real-time RT–PCR are listed in Supplementary Table S1 available at *JXB* online. Data are representative of two to three independent biological experiments.

### Bacterial growth assays

Five-week-old plants were spray inoculated with *Pseudomonas syringae* pv. *tomato* DC3000 (*Pst.* DC3000), and bacterial growth *in planta* was analysed as described by Zipfel *et al.* (2004). The bacterial suspension contained 2.5×10^8^ (Fig. 4A) or 2.5×10^6^ (Fig. 4B) colony-forming units (cfu) ml^–1^ in 10mM MgCl_2_ with 0.01% Silwet L-77. Eight plants of each genotype were used per experiment, and the experiments were repeated at least three times. Bacterial numbers in mutant and transgenic lines were compared with those in Col-0 using a two-tailed Student’s *t*-test.

### ROS burst assays

Flg22 peptide (QRLSTGSRINSAKDDAAGLQIA) and elf18 peptide (acetyl-MSKEKFERTKPHVNVGTI) were synthesized by GenScript Corp. Chitin (Seikagaku) was kindly provided by Dr Morten Petersen. All the above PAMPs were dissolved in sterile water. PAMP-induced ROS production was measured as previously described (Roux *et al.*, 2011; Schwessinger *et al.*, 2011). Briefly, leaf discs (0.125cm^2^) of 5-week-old plants were incubated overnight in 96-well plates in water, and the water was replaced with 200 μl of a solution containing 10 μg ml^–1^ peroxidase (Sigma-Aldrich) and 20 μM luminol in the presence of 100nM flg22, 100nM elf18, or 100 μg ml^–1^ chitin. Luminescence is shown as relative light units (RLUs), measured and calculated using a Berthold Centro LB960 luminometer (Berthhold Technologies).

### Callose deposition assays

Leaves of 5-week-old *Arabidopsis* plants were infiltrated with 1 μM flg22 with a needleless syringe. After 16h, the leaves were cleared, stained with aniline blue as previously described (Hann and Rathjen, 2007), mounted in 50% glycerol, and examined with a UV epifluorescence microscope. The numbers of bright spots (corresponding to callose deposits) per microscopic field of 1mm^2^ were counted using Image J software (http://imagej.nih.gov/ij/), and 12 microscopic fields were counted per sample.

### Root growth assays

*Arabidopsis* seeds were surface-sterilized and sown on 1/2 MS agar medium. Seeds were stratified at 4 °C for 2 d and grown vertically for 5 d in short-day conditions (8h light/16h dark). Seedlings were then transferred to a new square Petri dish with 1/2 MS agar medium supplemented with different amounts of flg22 peptide. The lengths of the main roots after growth under long-day conditions (16h light/8h dark) were measured with Image J software.

### Seedling growth assays

The seedling growth assays were performed as described (Pfund *et al.*, 2004). The fresh weight of seedlings was measured 8 d after flg22 treatment.

### Accession numbers

Sequence data from this article can be found in the *Arabidopsis* Genome Initiative or GenBank/EMBL databases under the following accession numbers: *Arabidopsis AtCLCa* (At5g40890), *AtCLCb* (At3g27170), *AtCLCc* (At5g49890), *AtCLCd* (At5g26240), *AtCLCe* (At4g35440), *AtCLCf* (At1g55620), *AtCLCg* (At5g33280), *FRK1* (At2g19190), *FLS2* (At5g46330), *PR1* (At2g14610), *ACTIN7* (At5g09810), and *ACTIN2* (At3g18780).

## Results

### *Arabidopsis clcd* mutant exhibits enhanced flg22-induced responses

**Fig. 1.**
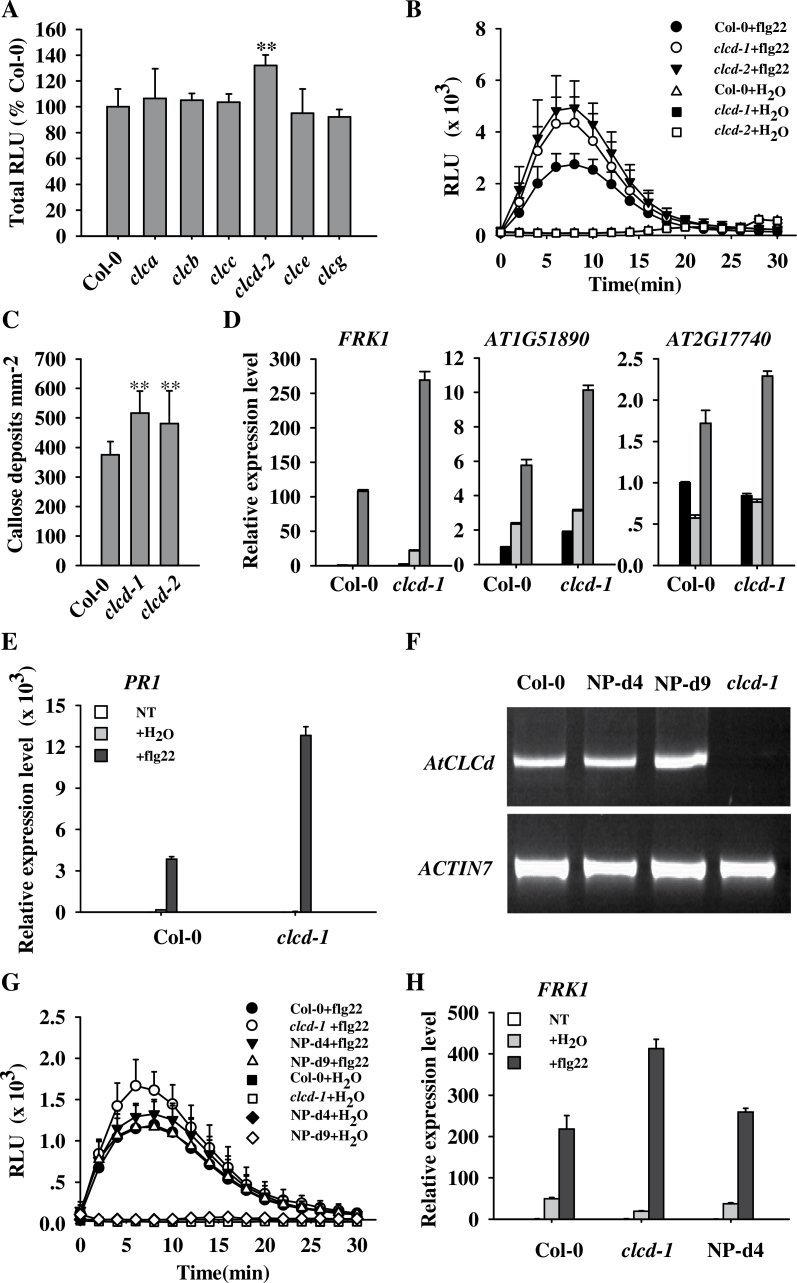
PAMP-triggered immunity is enhanced in *Arabidopsis clcd* mutants. (A) Total ROS production triggered by 100nM flg22 in *Arabidopsis* leaf discs in relative light units (RLU). Results are expressed as percentages of ﬂg22-treated Col-0. (B) flg22-induced ROS bursts in Col-0, *clcd-1*, and *clcd-2* leaf discs. (C) Callose deposition in Col-0, *clcd-1*, and *clcd-2* leaves after infiltration with 1 μM flg22 (*n*=22). (D and E) Quantitative real-time PCR analysis of the expression of PTI marker genes (D) and *PR1* (E) in *Arabidopsis* leaves 1h (D) and 24h (E) after infiltration with 1 μM flg22 or water. The samples were measured in triplicate and normalized to *ACTIN2*. (F) Expression of *AtCLCd* determined by semi-quantitative RT–PCR in the wild-type (Col-0), *AtCLCd* complementation lines (NP-d4 and NP-d9), and *clcd-1*. Levels of *ACTIN7* transcripts were used as loading controls. (G) Flg22-induced ROS bursts in Col-0, *clcd-1*, NP-d4, and NP-d9 in RLU. (H) Quantitative real-time PCR analysis of the expression of the PTI marker gene *FRK1* in Col-0, *clcd-1*, and NP-d4 plants 1h after infiltration with 1 μM flg22 or water. Samples were assayed in triplicate and normalized to *ACTIN2*. Values are means ±SD. NT, no treatment. **P<0.01 (*t*-test).

In a search for the anion channel(s) involved in plant defence responses, especially PTI, all available mutants of the *Arabidopsis CLC* gene family were screened using flg22-induced ROS as an indicator. flg22, a conserved 22 amino acid sequence at the N-terminus of flagellin, is a well-studied PAMP in plant innate immunity (Felix *et al.*, 1999; Bauer *et al.*, 2001). ROS production triggered by flg22 was measured in *Arabidopsis* T-DNA insertion lines of the *AtCLCa*, *b*, *c*, *d*, *e*, and *g* genes (Supplementary Fig. S1 available at *JXB* online). Leaf discs from all these lines except the *clcd* mutant produced similar ROS bursts to those produced by Col-0 (wild-type) *Arabidopsis* plants (Fig. 1A). However, the flg22-triggered ROS burst was significantly larger in the *clcd-2* mutant (Fig. 1A). This result was confirmed with another *clcd* mutant, *clcd-1* (Fig. 1B). The impact of *AtCLCd* mutations on other temporal responses triggered by flg22 was then examined (Roux *et al.*, 2011). The ROS burst is an early response to PAMPs, whereas callose deposition is a late response, detected in *Arabidopsis* by aniline blue staining ~16h after flg22 treatment (Boller and Felix, 2009). The flg22-induced callose deposition was also larger in *clcd-1* and *clcd-2* than in Col-0 (Fig. 1C). These findings indicate that AtCLCd is involved in PTI. Since the *clcd-1* and *clcd-2* mutants had almost identical phenotypes, to simplify the work, further analyses concentrated on the *clcd-1* mutant.

The expression of PAMP-inducible genes in the *clcd* mutants was next assessed. The expression levels of three different PTI marker genes *FRK1*, *At1g51890*, and *At2g17740* (He *et al.*, 2006) were measured 1h after infiltration with 1 μM flg22. As shown in Fig. 1D, the induction of all the three PTI marker genes was enhanced in the *clcd-1* mutant. Interestingly, the late response gene *PR1* was also induced to a strikingly higher level in *clcd-1* than in Col-0 (Fig. 1E).

In order to confirm that the phenotype of the *clcd* mutants is actually caused by mutation in the *AtCLCd* gene, *clcd-1* plants were transformed via *A. tumefaciens* with *pAtCLCd:AtCLCd*, a construct carrying the full-length open reading frame of *AtCLCd* driven by its own promoter (1571bp upstream of the start codon). A number of transgenic plants were obtained, most of which had the wild-type level of *AtCLCd* expression as checked by semi-quantitative RT–PCR (Fig. 1F). Representative lines homozygous for the rescue construct were used for further phenotypic analysis. The changes in the flg22-triggered ROS burst (Fig. 1G) and in the expression of *FRK1* (Fig. 1H) were also rescued by expressing *AtCLCd*.

In summary, it was shown that mutations in *AtCLCd* lead to enhanced early and late responses to flg22, suggesting that AtCLCd negatively regulates PTI.

### Overexpression of *AtCLCd* leads to impaired flg22-induced responses

**Fig. 2.**
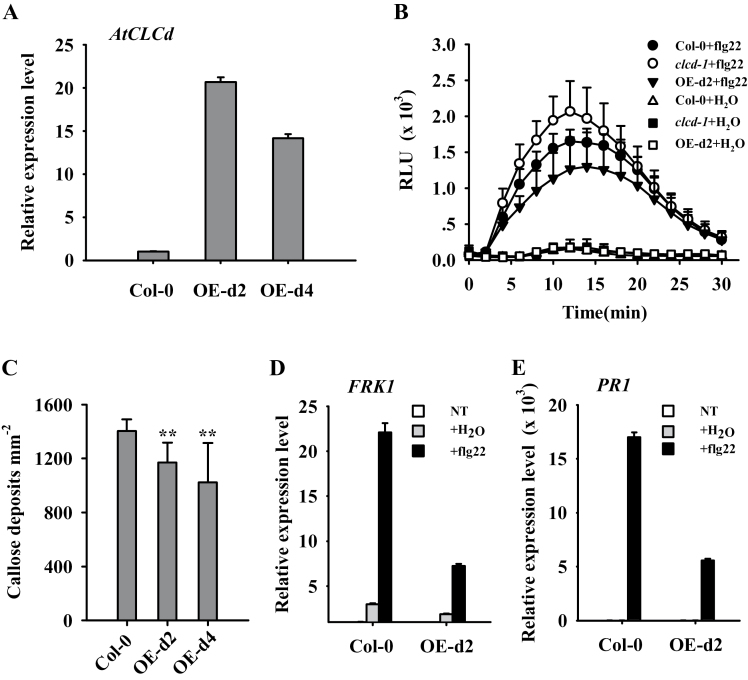
PAMP-triggered immunity is compromised in the *AtCLCd*-overexpressing lines. (A) Expression of *AtCLCd* was determined by quantitative RT–PCR in the wild type (Col-0) and *AtCLCd*-overexpressing lines (OE-d2 and OE-d4). (B) Flg22-induced ROS bursts in Col-0, *clcd-1*, and OE-d2 were measured in relative light units (RLU). Values are mean ±SD (*n*=8). (C) Flg22-induced callose deposition in the leaves of Col-0, OE-d2, and OE-d4 plants. Values are mean ±SD (*n*=12). **P<0.01 (*t*-test). (D and E) Quantitative real-time PCR analyses of the expression of *FRK1* (D) and *PR1* (E) in Col-0 and *AtCLCd*-overexpressing lines. Samples were assayed in triplicate and normalized to *ACTIN2*. Error bars indicate the SD. NT, no treatment.

To establish further the role of AtCLCd in PTI, transgenic plants constitutively overexpressing *AtCLCd* were created (see the Materials and methods). The full-length *AtCLCd* coding region driven by the CaMV 35S promoter was cloned into a binary vector and introduced into Col-0 plants. Two representative transgenic lines homozygous for the transgene (OE-d2 and OE-d4) with elevated *AtCLCd* transcript levels (Fig. 2A) were used for detailed phenotypic analyses. ROS bursts (Fig. 2B; Supplementary Fig. S2A available at *JXB* online) and callose deposition (Fig. 2C) triggered by flg22 were reduced in the *AtCLCd-*overexpressing lines. In addition, flg22-induced expression of the PTI marker genes *FRK1*, *At1g51890*, and *At2g17740*, and the late response gene *PR1* was impaired (Fig. 2D, E; Supplementary Fig. S2B available at JXB online). These data further demonstrate that AtCLCd is a negative regulator of PTI.

### Misexpression of *AtCLCd* affects PTI responses

**Fig. 3.**
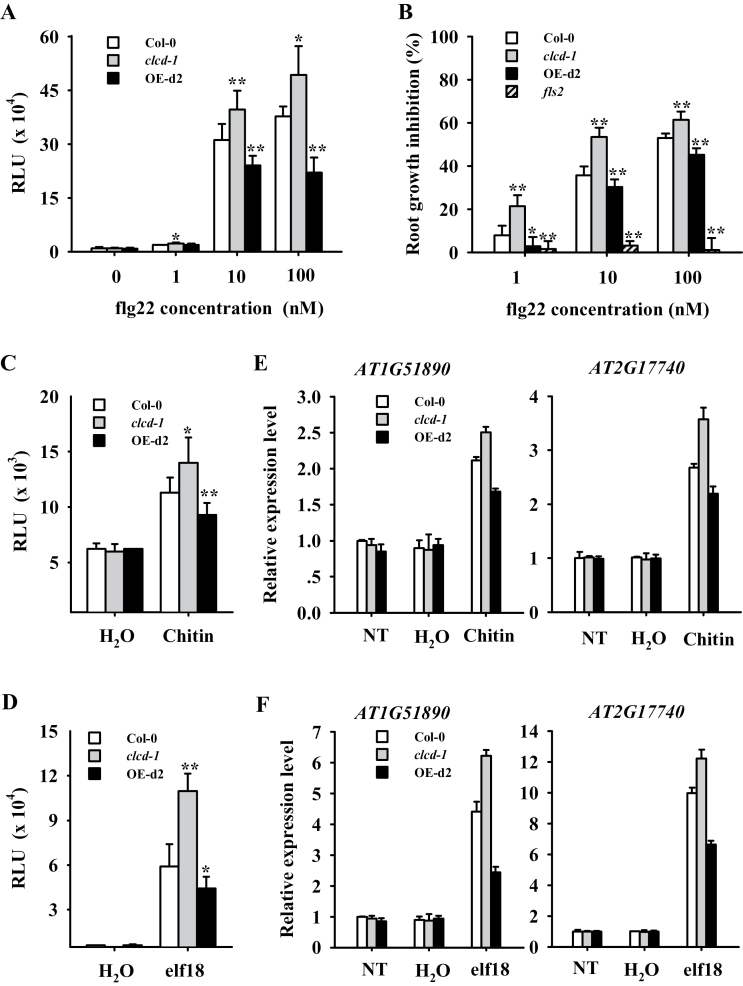
PAMP-triggered immunity is affected by misexpression of *AtCLCd*. (A) Total ROS production elicited by different amounts of flg22 in *Arabidopsis* leaf discs is represented as relative light units (RLU). Results are expressed as percentages of ﬂg22-treated Col-0, and are means ±SD (*n*=8). (B) Inhibition of primary root growth by different doses of flg22. Results are expressed as percentages of inhibition relative to the untreated control; means ±SD of three independent experiments (*n* >20). (C and D) Total ROS production induced by chitin or elf18 in Col-0, *clcd-1*, and OE-d2, measured in RLU. Values are means ±SD (*n*=8). (E and F) Quantitative RT–PCR analysis of the expression of the PTI marker genes 1h after treatment with chitin and elf18. NT, no treatment. *P<0.05; **P<0.01 (*t*-test).

To confirm the regulatory role of AtCLCd in PTI, the phenotypes of *AtCLCd-*misexpressing plants were investigated in more detail. The responses of the *clcd* mutants and the *AtCLCd*-overexpressing lines to various doses of flg22 were first analysed. As shown in Fig. 3A, production of ROS induced by flg22 was increased in the *clcd* mutant and reduced in the *AtCLCd-*overexpressing lines compared with the Col-0 wild type. Root growth inhibition is another characteristic effect of flg22 treatment (Boller and Felix, 2009). The *clcd-1* seedlings exhibited increased root growth inhibition in the presence of flg22, while root growth was less inhibited by flg22 in the *AtCLCd*-overexpressing lines (Fig. 3B). However, the most striking difference between the *AtCLCd*-misexpressing plants and the wild type in their responses to flg22 was observed after treating them with a lower dose (10nM) of the peptide (Fig. 3A, B). It is possible that a saturating dose of flg22 prevents detection of a partial effect of the mutation or of overexpression of *AtCLCd*. The effect of *AtCLCd* misexpression on flg22-triggered responses was further revealed by seedling growth inhibition assays (Supplementary Fig. S3 available at *JXB* online).

Responses of the *AtCLCd-*misexpressing plants to different PAMPs were next examined. Production of ROS elicited by both chitin and elf18 was significantly enhanced in the *clcd* mutant, but reduced in the *AtCLCd*-overexpressing lines (Fig. 3C, D). Accordingly, expression of the PTI marker genes *At1g51890* and *At2g17740* was also increased in the mutant, but decreased in the overexpressing lines (Fig. 3E, F). In addition, the morphological phenotypes and expression levels of *FLS2* (Supplementary Fig. S4 available at *JXB* online) were not changed in the *AtCLCd*-misexpressing plants. These results support a general role for AtCLCd in PTI.

### Altered bacterial disease resistance in *Arabidopsis clcd* mutants and *AtCLCd-*overexpressing plants

**Fig. 4.**
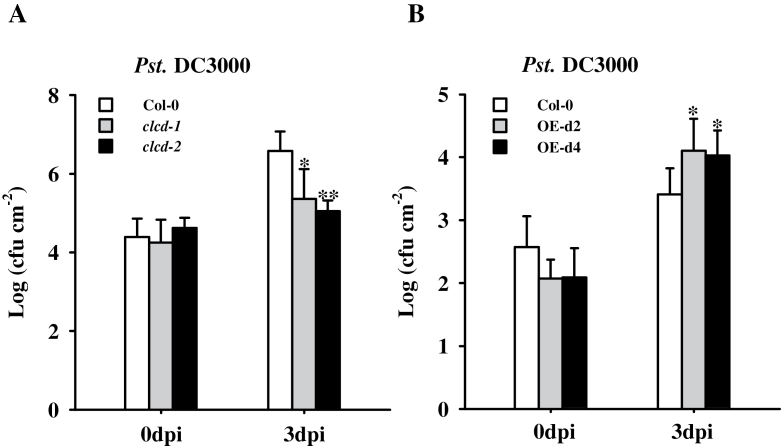
Responses of the *clcd* mutants and *AtCLCd*-overexpressing lines to the pathogen *Pseudomonas syringae*. (A and B) Bacterial growth of *Pst.* DC3000 was measured in Col-0, *clcd-1*, and *clcd-2* (A) or Col-0 and *AtCLCd*-overexpressing lines OE-d2 and OE-d4 (B) on 0 d and 3 d post spray inoculation (dpi). Bacterial suspensions containing 2.5×10^8^ (A) and 2.5×10^6^ (B) cfu ml^–1^ were used. Values are mean ±SD (*n*=8). *P<0.05; **P<0.01 (*t*-test). cfu, colony-forming units.

PTI plays an important role in basal resistance to bacterial pathogens (Jones and Dangl, 2006), and defects in PTI, due to mutations in the PRR FLS2 and its positive regulator BAK1, result in enhanced susceptibility to the virulent bacterial pathogen *Pst.* DC3000 upon spray inoculation (Zipfel *et al.*, 2004; Roux *et al.*, 2011). Tests were carried out to determine whether AtCLCd controls disease resistance. Four-week-old *clcd-1* and *clcd-2* plants were spray inoculated with *Pst*. DC3000, and growth of the bacterial pathogen in the leaves was assessed. As shown in Fig. 4A, growth of *Pst.* DC3000 in the leaves of the *clcd* mutants was reduced, whereas in the *AtCLCd*-overexpressing lines it was increased (Fig. 4B). These data further support the inhibitory role of AtCLCd in PTI.

### Treatment with the PAMP, flg22, represses the expression of *AtCLCd*

**Fig. 5.**
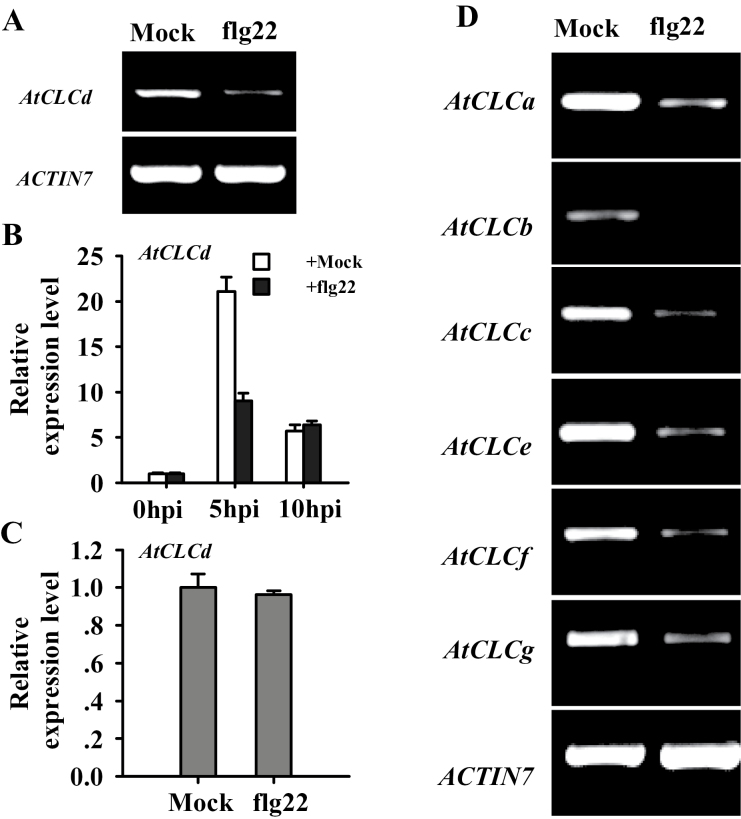
Treatment with PAMP flg22 inhibits the expression of *Arabidopsis CLC* family genes. (A) Expression of *AtCLCd* in water- and flg22-treated wild-type (Col-0) leaves. (B) Accumulation of *AtCLCd* transcripts in Col-0 leaves 5h and 10h after infiltration of 1 μM flg22 or water. (C) Expression of *AtCLCd* in *fls2* mutant leaves 5h after infiltration of 1 μM flg22 or water. (D) Expression of *AtCLC* family genes in water- and flg22-treated Col-0 samples 5h after infiltration. Semi-quantitative RT–PCR was performed in (A) and (D). The level of *ACTIN7* transcript was used as a loading control. Quantitative RT–PCR was performed in (B) and (C). All samples were assayed in triplicate and normalized to *ACTIN2*. In (C), the relative expression ratio of *AtCLCd* transcript was compared with that of water-treated *fls2* plants, which are set to a relative value of 1. Error bars indicate the SD. hpi, hours post-infiltration.

Since AtCLCd negatively regulates PTI, it was of interest to see whether *AtCLCd* expression was affected by PAMPs. To this end, 4-week-old Col-0 plants grown under short-day conditions were infiltrated with 1 μM flg22 or water (mock). The expression of *AtCLCd* was reduced by the flg22 treatment (Fig. 5A). Expression of *AtCLCd* was then examined in more detail (Fig. 5B). Treatment with water (as a control) stimulated the expression of *AtCLCd* (Fig. 5B), probably due to damage introduced by the infiltration. However, compared with the water-treated plants, the accumulation of *AtCLCd* mRNA was significantly reduced in the flg22-treated plants at 5h post-treatment (Fig. 5B), further showing that flg22 negatively regulates the expression of *AtCLCd*. By 10h, expression of *AtCLCd* was similar in the flg22-treated and water-treated plants. To see whether the repression of *AtCLCd* by flg22 is dependent on FLS2, expression of *AtCLCd* was measured in *fls2* mutant plants (SALK_141277, Xiang *et al.*, 2008) infiltrated with 1 μM flg22 or water. As shown in Fig. 5C, transcripts of *AtCLCd* accumulated to similar level in flg22-treated plants and mock-treated plants, indicating that flg22 does not suppress the expression of *AtCLCd* in the *fls2* mutant. Thus, it can be concluded that recognition of flg22 by FLS2 is needed for the inhibition of *AtCLCd* expression.

Next, the response of all other *Arabidopsis CLC* family genes to flg22 was examined. Interestingly, all were affected in the same way as *AtCLCd* (Fig. 5D). It appears therefore that the sensitivity of expression to flg22 may be common to all *Arabidopsis CLC* genes.

### FLS2 signalling complexes regulate the expression of *AtCLCd*

**Fig. 6.**
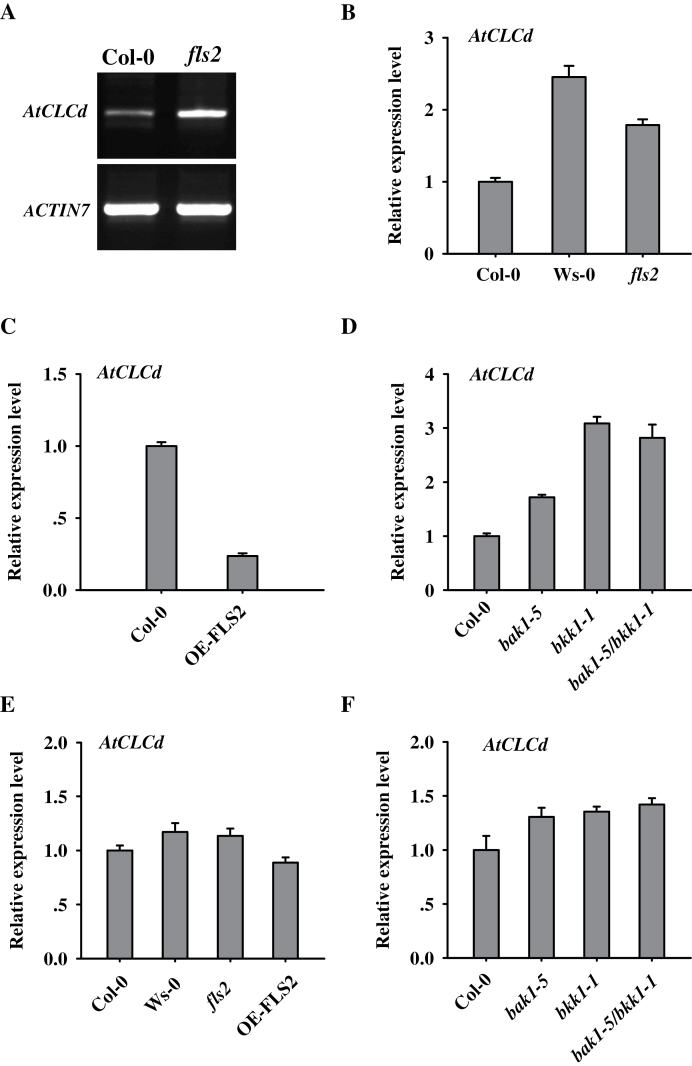
FLS2 and its signalling partners participate in regulating *AtCLCd* gene expression. (A) Expression of *AtCLCd* in Col-0 and *fls2* mutant leaves analysed by semi-quantitative PCR. The level of *ACTIN7* transcript was used as a loading control. (B–D) Expression of *AtCLCd* determined by quantitative RT–PCR in Col-0, Ws-0, and *fls2* plants (B), in Col-0 and an *FLS2*-overexpressing line (OE-FLS2) (C), and in Col-0, *bak1-5*, *bkk1-1*, and *bak1-5/bkk1-1* plants (D). (E and F) Expression levels of *AtCLCd* in Col-0, Ws-0, *fls2*, OE-FLS2 (E); and *bak1-5*, *bkk1-1*, and *bak1-5*/*bkk1-1* (F) 14-day-old seedlings grown under sterile conditions. All samples were assayed in triplicate and normalized to *ACTIN2*. Error bars indicate the SD.

It was noticed that expression of *AtCLCd* was higher in the *fls2* mutant than in Col-0 (Fig. 6A). To confirm this finding, transcript levels in the *fls2* mutant and the Wassilewskija (Ws-0) background were compared. The ecotype Ws-0 is a natural *fls2* mutant (Gómez-Gómez and Boller, 2000; Zipfel *et al.*, 2004). As shown in Fig. 6B, *AtCLCd* transcript levels in the Ws-0 background were similar to those in the *fls2* mutant and almost 2- to 2.5-fold higher than in Col-0. Moreover, *AtCLCd* transcript levels were strikingly reduced in the *FLS2*-overexpressing line (Fig. 6C). These results indicate that PRR FLS2 negatively regulates the expression of *AtCLCd*.

*Arabidopsis* somatic embryogenesis receptor-like kinases (SERKs) form complexes with PRRs in a ligand-dependent manner (Monaghan and Zipfel, 2012). BAK1/SERK3 and BKK1/SERK4 are required for FLS2-mediated PTI signalling in *Arabidopsis* (Roux *et al.*, 2011). Recently, a novel *bak1-5* mutant allele was identified in which only PTI was impaired, thereby avoiding the pleiotropic effects of the other *bak1* mutations (Schwessinger *et al.*, 2011). Even though the *bkk1-1* mutant exhibited wild-type-like responses to flg22, loss of BKK1 further decreased the early and late responses of *bak1-5* to flg22 (Roux *et al.*, 2011). The study was thus extended to quantify expression of *AtCLCd* in *bak1-5*, *bkk1-1*, and a *bak1-5*/*bkk1-1* double mutant. As shown in Fig. 6D, transcripts of *AtCLCd* accumulated to higher levels in the three mutants than in Col-0. Expression of *AtCLCd* was higher in *bkk1-1* than in *bak1-5*. The combination of the two mutations had no additive effect on expression (Fig. 6D). These findings imply that the FLS2 regulatory proteins BAK1 and BKK1 play a role in the regulation of *AtCLCd* expression and that they function in the same pathway.

Plants are always exposed to a variety of microbes under non-sterile soil conditions; therefore, *AtCLCd* expression in sterile seedlings was checked. As shown in Fig. 6E, the accumulation of *AtCLCd* transcripts did not change significantly in Col-0, Ws-0, *fls2*, and *FLS2*-overexpressing plants under sterile conditions. Interestingly, expression of *AtCLCd* in *bak1-5*, *bkk1-1*, and *bak1-5*/*bkk1-1* plants was also significantly reduced under sterile conditions, but was still a little higher than that in wild-type plants (Fig. 6F). These results support that PAMP perception is required for FLS2 to regulate *AtCLCd* expression.

Taken together, the above results indicate that the FLS2 signalling complex regulates the expression of *AtCLCd* in *Arabidopsis*.

## Discussion

Changes in cellular anion content are thought to be associated with plant defence responses (De Angeli *et al.*, 2007; Gauthier *et al.*, 2007; Errakhi *et al.*, 2008; Colcombet *et al.*, 2009). *Arabidopsis CLC* family genes encode putative anion channels (Barbier-Brygoo *et al.*, 2011). However, whether CLC channels participate in plant innate immunity was still unclear. Therefore, T-DNA insertion lines of *AtCLCa*, *b*, *c*, *d*, *e*, and *g* were screened for changes in flg22-induced ROS, and it was found that *clcd* mutants were unique in displaying enhanced ROS production in response to flg22 (Fig. 1). This and other findings (Figs 2, 3) provide ample evidence that AtCLCd is a negative regulator of PTI.

It was further shown that the PAMP, flg22, represses the expression of *AtCLCd* (Fig. 5A, B). However, the repression was not seen in a mutant of the flg22 receptor FLS2 (Fig. 5C). This and other findings (Fig. 6) showed that the FLS2 complex is required for maintaining the expression of *AtCLCd*. Since expression of *FLS2* is induced by flg22 (Zipfel *et al.*, 2004), it is possible that the decrease in expression of *AtCLCd* by flg22 is due in part to an increase of FLS2. Expression of the other *Arabidopsis CLC* genes was also reduced upon treatment with flg22 (Fig 5D), suggesting that these genes also play a role in PTI. However, ROS production was unchanged in the corresponding mutants (Fig. 1). A possible explanation for that is that there is functional redundancy of these genes. When compared with *CLC* genes from the monocot, rice, *AtCLCd* forms its own group, whereas the other *AtCLC* genes cluster together (von der Fecht-Bartenbach *et al.*, 2010). Analysis of combinations of these mutants may be necessary to elucidate their roles in plant innate immunity.

AtCLCd has been shown to localize to the TGN (von der Fecht-Bartenbach *et al.*, 2007; Lv *et al.*, 2009). Therefore, it is unlikely that it is directly involved in downstream signalling upon PAMP perception, which takes place at the plasma membrane. The yeast genome encodes only one CLC protein, Gef1p, and it regulates the intra-Golgi pH (Hechenberger *et al.*, 1996). Expression of *AtClCd* fully rescues the *gef1* yeast mutant phenotype (Hechenberger *et al*., 1996; Marmagne *et al*., 2007; Lv *et al.*, 2009), suggesting that AtClCd may have a function similar to Gef1p. Moreover, AtClCd has been shown to co-localize with the V-type ATPase subunit, VHA-a1, in the TGN (von der Fecht-Bartenbach *et al.*, 2007). Inhibition of VHA-a1 affects Golgi morphology and restricts cell expansion (Dettmer *et al.*, 2005, 2006; Brüx *et al.*, 2008), and this effect is enhanced in the *clcd* mutant (von der Fecht-Bartenbach *et al.*, 2007), further implying that AtCLCd is involved in adjusting the luminal pH of the TGN. pH homeostasis of the TGN is essential for its functioning (Demaurex *et al.*, 1998; Dettmer *et al.*, 2006). Therefore, AtCLCd most probably regulates the functioning of the TGN by affecting the pH within it.

The TGN is an important platform for sorting cargo proteins to the cell surface or vacuole and lysosome (Viotti *et al.*, 2010; Beck *et al.*, 2012a). A distinctive characteristic of the membrane trafficking system in plants is the convergence of the secretory and endocytic pathways at the TGN (Dettmer *et al.*, 2006; Dhonukshe *et al.*, 2007; Viotti *et al.*, 2010). Endocytic membrane transport has been observed for several plasma membrane receptors in plants, and this seems to be a general regulatory mechanism for perception of extracellular stimuli by plasma membrane receptors (Takano *et al.*, 2002; Robatzek *et al.*, 2006; Beck *et al.*, 2012a). FLS2, the PRR for flg22 (Gómez-Gómez and Boller, 2000, 2002), is localized to the plasma membrane and becomes specifically internalized into highly mobile vesicles upon addition of flg22 (Robatzek *et al.*, 2006). The endocytic transport of FLS2 is critical for its function in PTI (Robatzek *et al.*, 2006). Recently, it was shown that the TGN is an essential compartment for membrane trafficking of FLS2 (Beck *et al.*, 2012b; Choi *et al.*, 2013; Uemura and Nakano, 2013). Interestingly, AtCLCd has been previously suggested to be involved in membrane trafficking, since AtCLCd–green fluorescent protein (GFP) co-localized with endocytosed FM4-64, a dye widely used for tracing endocytic membrane traffic (von der Fecht-Bartenbach *et al.*, 2007). It thus seems likely that AtCLCd regulates PTI via the TGN, probably by affecting FLS2 trafficking. Endocytosis is a feature of most of the PRRs in plants (Beck, *et al.*, 2012a). In agreement with this, it was also found here that different PAMP-induced defence responses were impaired in the *AtCLCd-*misexpressing plants (Fig. 3). Nevertheless, further studies are needed to reveal whether FLS2 trafficking is affected in the *clcd* mutant and the overexpressing lines.

In summary, it has been shown here that AtCLCd negatively regulates *Arabidopsis* PTI, probably by interacting with the PRR signalling pathway. Its sequence indicates that *AtCLCd* encodes a chloride/proton exchanger (Zifarelli and Pusch, 2010). Future work involving classical and patch–clamp electrophysiology should establish whether the role of AtCLCd in PTI requires a functional channel.

## Supplementary data

Supplementary data are available at *JXB* online.

Table S1. Sequences of primers used in this work

Figure S1. Characterization of *Arabidopsis CLC* T-DNA insertion lines.

Figure S2. PAMP-triggered immunity is compromised in the *AtCLCd*-overexpressing lines.

Figure S3. Shoot growth inhibition induced by flg22 in Col-0, *clcd* mutant and *AtCLCd-*overexpressing plants.

Figure S4. Morphological phenotypes and *FLS2* expression levels in the *AtCLCd-*misexpressing plants.

Supplementary Data

## References

[CIT0001] Barbier-Brygoo H De Angeli A Filleur S Frachisse JM Gambale F Thomine S Wege S 2011 Anion channels/transporters in plants: from molecular bases to regulatory networks. Annual Review of Plant Biology 62, 25–51 10.1146/annurev-arplant-042110-103741 21275645

[CIT0002] Bauer Z Gómez-Gómez L Boller T Felix G 2001 Sensitivity of different ecotypes and mutants of *Arabidopsis thaliana* toward the bacterial elicitor flagellin correlates with the presence of receptor-binding sites. Journal of Biological Chemistry 276, 45669–45676 11564731 10.1074/jbc.M102390200

[CIT0003] Beck M Heard W Mbengue M Robatzek S 2012a The INs and OUTs of pattern recognition receptors at the cell surface. Current Opinion in Plant Biology 15, 367–374 22664220 10.1016/j.pbi.2012.05.004

[CIT0004] Beck M Zhou J Faulkner C MacLean D Robatzek S 2012b Spatio-temporal cellular dynamics of the *Arabidopsis* flagellin receptor reveal activation status-dependent endosomal sorting. The Plant Cell 24, 4205–4219 23085733 10.1105/tpc.112.100263 PMC3516521

[CIT0005] Boller T Felix G 2009 A renaissance of elicitors: perception of microbe-associated molecular patterns and danger signals by pattern-recognition receptors. Annual Review of Plant Biology 60, 379–406 10.1146/annurev.arplant.57.032905.105346 19400727

[CIT0006] Boller T He SY 2009 Innate immunity in plants: an arms race between pattern recognition receptors in plants and effectors in microbial pathogens. Science 324, 742 19423812 10.1126/science.1171647 PMC2729760

[CIT0007] Brüx A Liu TY Krebs M Stierhof YD Lohmann JU Miersch O Wasternack C Schumacher K 2008 Reduced V-ATPase activity in the trans-Golgi network causes oxylipin-dependent hypocotyl growth inhibition in *Arabidopsis* . The Plant Cell 20, 1088–1100 18441211 10.1105/tpc.108.058362 PMC2390726

[CIT0008] Choi SW Tamaki T Ebine K Uemura T Ueda T Nakano A 2013 RABA members act in distinct steps of subcellular trafficking of the FLAGELLIN SENSING2 receptor. The Plant Cell 25, 1174–1187 23532067 10.1105/tpc.112.108803 PMC3634684

[CIT0009] Clough SJ Bent AF 1998 Floral dip: a simplified method for *Agrobacterium*-mediated transformation of *Arabidopsis thaliana* . The Plant Journal 16, 735–743 10069079 10.1046/j.1365-313x.1998.00343.x

[CIT0010] Colcombet J Mathieu Y Peyronnet R Agier N Lelièvre F Barbier-Brygoo H Frachisse JM 2009 R-type anion channel activation is an essential step for ROS-dependent innate immune response in *Arabidopsis* suspension cells. Functional Plant Biology 36, 832–843 10.1071/FP09096 32688693

[CIT0011] De Angeli A Thomine S Frachisse JM Ephritikhine G Gambale F Barbier-Brygoo H 2007 Anion channels and transporters in plant cell membranes. FEBS Letters 581, 2367–2374 17434490 10.1016/j.febslet.2007.04.003

[CIT0012] Demaurex N Furuya W D’Souza S Bonifacino JS Grinstein S 1998 Mechanism of acidification of the trans-Golgi network (TGN). *In situ* measurements of pH using retrieval of TGN38 and furin from the cell surface. Journal of Biological Chemistry 273, 2044–2051 9442042 10.1074/jbc.273.4.2044

[CIT0013] Dettmer J Hong-Hermesdorf A Stierhof YD Schumacher K 2006 Vacuolar H^+^-ATPase activity is required for endocytic and secretory trafficking in *Arabidopsis* . The Plant Cell 18, 715–730 16461582 10.1105/tpc.105.037978 PMC1383645

[CIT0014] Dettmer J Schubert D Calvo-Weimar O Stierhof YD Schmidt R Schumacher K 2005 Essential role of the V-ATPase in male gametophyte development. The Plant Journal 41, 117–124 15610354 10.1111/j.1365-313X.2004.02282.x

[CIT0015] Dhonukshe P Aniento F Hwang I Robinson DG Mravec J Stierhof YD Friml J 2007 Clathrin-mediated constitutive endocytosis of PIN auxin efflux carriers in *Arabidopsis* . Current Biology 17, 520–527 17306539 10.1016/j.cub.2007.01.052

[CIT0016] Errakhi R Meimoun P Lehner A Vidal G Briand J Corbineau F Rona JP Bouteau F 2008 Anion channel activity is necessary to induce ethylene synthesis and programmed cell death in response to oxalic acid. Journal of Experimental Botany 59, 3121–3129 18612171 10.1093/jxb/ern166

[CIT0017] Felix G Duran JD Volko S Boller T 1999 Plants have a sensitive perception system for the most conserved domain of bacterial flagellin. The Plant Journal 18, 265–276 10377992 10.1046/j.1365-313x.1999.00265.x

[CIT0018] Gauthier A Lamotte O Reboutier D Bouteau F Pugin A Wendehenne D 2007 Cryptogein-induced anion effluxes: electrophysiological properties and analysis of the mechanisms through which they contribute to the elicitor-triggered cell death. Plant Signaling and Behavior 2, 86–95 19516973 10.4161/psb.2.2.4015 PMC2633904

[CIT0019] Gómez-Gómez L Boller T 2000 FLS2: an LRR receptor-like kinase involved in the perception of the bacterial elicitor flagellin in *Arabidopsis* . Molecular Cell 5, 1003–1011 10911994 10.1016/s1097-2765(00)80265-8

[CIT0020] Gómez-Gómez L Boller T 2002 Flagellin perception: a paradigm for innate immunity. Trends in Plant Science 7, 251–256 12049921 10.1016/s1360-1385(02)02261-6

[CIT0021] Hann DR Rathjen JP 2007 Early events in the pathogenicity of *Pseudomonas syringae* on *Nicotiana benthamiana* . The Plant Journal 49, 607–618 17217460 10.1111/j.1365-313X.2006.02981.x

[CIT0022] He P Shan L Lin NC Martin GB Kemmerling B Nürnberger T Sheen J 2006 Specific bacterial suppressors of MAMP signaling upstream of MAPKKK in *Arabidopsis* innate immunity. Cell 125, 563–575 16678099 10.1016/j.cell.2006.02.047

[CIT0023] Hechenberger M Schwappach B Fischer WN Frommer WB Jentsch TJ Steinmeyer K 1996 A family of putative chloride channels from *Arabidopsis* and functional complementation of a yeast strain with a *CLC* gene disruption. Journal of Biological Chemistry 271, 33632–33638 8969232 10.1074/jbc.271.52.33632

[CIT0024] Jabs T Tschöpe M Colling C Hahlbrock K Scheel D 1997 Elicitor-stimulated ion fluxes and O^2−^ from the oxidative burst are essential components in triggering defense gene activation and phytoalexin synthesis in parsley. Proceedings of the National Academy of Sciences, USA 94, 4800–4805 10.1073/pnas.94.9.4800 PMC20805 9114072

[CIT0025] Jentsch TJ 2008 CLC chloride channels and transporters: from genes to protein structure, pathology and physiology. Critical Reviews in Biochemistry and Molecular Biology 43, 3–36 18307107 10.1080/10409230701829110

[CIT0026] Jeworutzki E Roelfsema MRG Anschütz U Krol E Elzenga JTM Felix G Boller T Hedrich R Becker D 2010 Early signaling through the *Arabidopsis* pattern recognition receptors FLS2 and EFR involves Ca^2+^-associated opening of plasma membrane anion channels. The Plant Journal 62, 367–378 20113440 10.1111/j.1365-313X.2010.04155.x

[CIT0027] Jones JD Dangl JL 2006 The plant immune system. Nature 444, 323–329 17108957 10.1038/nature05286

[CIT0028] Jossier M Kroniewicz L Dalmas F Le Thiec D Ephritikhine G Thomine S Barbier-Brygoo H Vavasseur A Filleur S Leonhardt N 2010 The *Arabidopsis* vacuolar anion transporter, AtCLCc, is involved in the regulation of stomatal movements and contributes to salt tolerance. The Plant Journal 64, 563–576 20822503 10.1111/j.1365-313X.2010.04352.x

[CIT0029] Koers S Guzel-Deger A Marten I Roelfsema MRG 2011 Barley mildew and its elicitor chitosan promote closed stomata by stimulating guard cell S-type anion channels. The Plant Journal 68, 670–680 21781196 10.1111/j.1365-313X.2011.04719.x

[CIT0030] Kunze G Zipfel C Robatzek S Niehaus K Boller T Felix G 2004 The N terminus of bacterial elongation factor Tu elicits innate immunity in *Arabidopsis* plants. The Plant Cell 16, 3496–3507 15548740 10.1105/tpc.104.026765 PMC535888

[CIT0031] Lv Q-D Tang Rj Liu H Gao Xs Li Yz Zheng Hq Zhang Hx 2009 Cloning and molecular analyses of the *Arabidopsis thaliana* chloride channel gene family. Plant Science 176, 650–661

[CIT0032] Marmagne A Vinauger-Douard M Monachello D de Longevialle AF Charon C Allot M Rappaport F Wollman FA Barbier-Brygoo H Ephritikhine G 2007 Two members of the *Arabidopsis* CLC (chloride channel) family, AtCLCe and AtCLCf, are associated with thylakoid and Golgi membranes, respectively. Journal of Experimental Botany 58, 3385–3393 17872921 10.1093/jxb/erm187

[CIT0033] Meyer S Mumm P Imes D Endler A Weder B Al-Rasheid KA Geiger D Marten I Martinoia E Hedrich R 2010 AtALMT12 represents an R-type anion channel required for stomatal movement in *Arabidopsis* guard cells. The Plant Journal 63, 1054–1062 20626656 10.1111/j.1365-313X.2010.04302.x

[CIT0034] Monaghan J Zipfel C 2012 Plant pattern recognition receptor complexes at the plasma membrane. Current Opinion in Plant Biology 15, 349–357 22705024 10.1016/j.pbi.2012.05.006

[CIT0035] Montillet JL Leonhardt N Mondy S Tranchimand S Rumeau D Boudsocq M Garcia AV Douki T Bigeard J Laurière C 2013 An abscisic acid-independent oxylipin pathway controls stomatal closure and immune defense in *Arabidopsis* . PLoS Biology 11, e1001513 23526882 10.1371/journal.pbio.1001513 PMC3602010

[CIT0036] Nürnberger T Nennstiel D Jabs T Sacks WR Hahlbrock K Scheel D 1994 High affinity binding of a fungal oligopeptide elicitor to parsley plasma membranes triggers multiple defense responses. Cell 78, 449–460 8062387 10.1016/0092-8674(94)90423-5

[CIT0037] Negi J Matsuda O Nagasawa T Oba Y Takahashi H Kawai-Yamada M Uchimiya H Hashimoto M Iba K 2008 CO_2_ regulator SLAC1 and its homologues are essential for anion homeostasis in plant cells. Nature 452, 483–486 18305482 10.1038/nature06720

[CIT0038] Pfund C Tans-Kersten J Dunning FM Alonso JM Ecker JR Allen C Bent AF 2004 Flagellin is not a major defense elicitor in Ralstonia solanacearum cells or extracts applied to Arabidopsis thaliana. Molecular Plant-Microbe Interactions 17, 696–706 15195952 10.1094/MPMI.2004.17.6.696

[CIT0039] Qi Z Verma R Gehring C Yamaguchi Y Zhao Y Ryan CA Berkowitz GA 2010 Ca^2+^ signaling by plant *Arabidopsis thaliana* Pep peptides depends on AtPepR1, a receptor with guanylyl cyclase activity, and cGMP-activated Ca^2+^ channels. Proceedings of the National Academy of Sciences, USA 107, 21193–21198 10.1073/pnas.1000191107 PMC3000296 21088220

[CIT0040] Robatzek S Chinchilla D Boller T 2006 Ligand-induced endocytosis of the pattern recognition receptor FLS2 in *Arabidopsis* . Genes and Development 20, 537–542 16510871 10.1101/gad.366506 PMC1410809

[CIT0041] Robatzek S Saijo Y 2008 Plant immunity from A to Z. Genome Biology 9, 304 18423060 10.1186/gb-2008-9-4-304 PMC2643932

[CIT0042] Roux M Schwessinger B Albrecht C Chinchilla D Jones A Holton N Malinovsky FG Tör M de Vries S Zipfel C 2011 The *Arabidopsis* leucine-rich repeat receptor-like kinases BAK1/SERK3 and BKK1/SERK4 are required for innate immunity to hemibiotrophic and biotrophic pathogens. The Plant Cell 23, 2440–2455 21693696 10.1105/tpc.111.084301 PMC3160018

[CIT0043] Saji S Bathula S Kubo A Tamaoki M Kanna M Aono M Nakajima N Nakaji T Takeda T Asayama M 2008 Disruption of a gene encoding C4-dicarboxylate transporter-like protein increases ozone sensitivity through deregulation of the stomatal response in *Arabidopsis thaliana* . Plant and Cell Physiology 49, 2–10 18084014 10.1093/pcp/pcm174

[CIT0044] Schroeder JI Keller BU 1992 Two types of anion channel currents in guard cells with distinct voltage regulation. Proceedings of the National Academy of Sciences, USA 89, 5025–5029 10.1073/pnas.89.11.5025 PMC49221 1375754

[CIT0045] Schwessinger B Roux M Kadota Y Ntoukakis V Sklenar J Jones A Zipfel C 2011 Phosphorylation-dependent differential regulation of plant growth, cell death, and innate immunity by the regulatory receptor-like kinase BAK1. PLoS Genetics 7, e1002046 21593986 10.1371/journal.pgen.1002046 PMC3085482

[CIT0046] Takano J Noguchi K Yasumori M Kobayashi M Gajdos Z Miwa K Hayashi H Yoneyama T Fujiwara T 2002 *Arabidopsis* boron transporter for xylem loading. Nature 420, 337–340 12447444 10.1038/nature01139

[CIT0047] Tsuda K Katagiri F 2010 Comparing signaling mechanisms engaged in pattern-triggered and effector-triggered immunity. Current Opinion in Plant Biology 13, 459–465 20471306 10.1016/j.pbi.2010.04.006

[CIT0048] Uemura T Nakano A 2013 Plant TGNs: dynamics and physiological functions. Histochemistry and Cell Biology 140, 341–345 23832380 10.1007/s00418-013-1116-7

[CIT0049] Vahisalu T Kollist H Wang YF Nishimura N Chan WY Valerio G Lamminmäki A Brosché M Moldau H Desikan R 2008 SLAC1 is required for plant guard cell S-type anion channel function in stomatal signalling. Nature 452, 487–491 18305484 10.1038/nature06608 PMC2858982

[CIT0050] Viotti C Bubeck J Stierhof YD Krebs M Langhans M van den Berg W van Dongen W Richter S Geldner N Takano J 2010 Endocytic and secretory traffic in *Arabidopsis* merge in the trans-Golgi network/early endosome, an independent and highly dynamic organelle. The Plant Cell 22, 1344–1357 20435907 10.1105/tpc.109.072637 PMC2879741

[CIT0051] von der Fecht-Bartenbach J Bogner M Dynowski M Ludewig U 2010 CLC-b-mediated NO^3^-/H^+^ exchange across the tonoplast of *Arabidopsis* vacuoles. Plant and Cell Physiology 51, 960–968 20430762 10.1093/pcp/pcq062

[CIT0052] von der Fecht-Bartenbach J Bogner M Krebs M Stierhof YD Schumacher K Ludewig U 2007 Function of the anion transporter AtCLC-d in the trans-Golgi network. The Plant Journal 50, 466–474 17376158 10.1111/j.1365-313X.2007.03061.x PMC1891005

[CIT0053] Wendehenne D Lamotte O Frachisse JM Barbier-Brygoo H Pugin A 2002 Nitrate efflux is an essential component of the cryptogein signaling pathway leading to defense responses and hypersensitive cell death in tobacco. The Plant Cell 14, 1937–1951 12172032 10.1105/tpc.002295 PMC151475

[CIT0054] Xiang C Han P Lutziger I Wang K Oliver DJ 1999 A mini binary vector series for plant transformation. Plant Molecular Biology 40, 711–717 10480394 10.1023/a:1006201910593

[CIT0055] Xiang T Zong N Zou Y Wu Y Zhang J Xing W Li Y Tang X Zhu L Chai J Zhou JM 2008 *Pseudomonas syringae* effector AvrPto blocks innate immunity by targeting receptor kinases. Current Biology 18, 74–80 18158241 10.1016/j.cub.2007.12.020

[CIT0056] Zifarelli G Pusch M 2010 CLC transport proteins in plants. FEBS Letters 584, 2122–2127 20036660 10.1016/j.febslet.2009.12.042

[CIT0057] Zipfel C Robatzek S Navarro L Oakeley EJ Jones JD Felix G Boller T 2004 Bacterial disease resistance in *Arabidopsis* through flagellin perception. Nature 428, 764–767 15085136 10.1038/nature02485

