# Supplementary material for: The chloride channel family gene CLCd negatively regulates pathogen-associated molecular pattern (PAMP)-triggered immunity in Arabidopsis
Source: J Exp Bot. 2014 Jan 21;65(4):1205–15. doi: 10.1093/jxb/ert484 (PMC3935575; doi:10.1093/jxb/ert484)
Supplement: Supplementary Data [file supp_65_4_1205__index.html]

The chloride channel family gene CLCd negatively regulates PAMP-triggered immunity in Arabidopsis — The chloride channel family gene CLCd negatively regulates pathogen-associated molecular pattern (PAMP)-triggered immunity in Arabidopsis — Supplementary Data 

# The chloride channel family gene *CLCd* negatively regulates pathogen-associated molecular pattern (PAMP)-triggered immunity in *Arabidopsis*

## Supplementary Data

Data files

**Files in this Data Supplement:**

- Supplementary Data - Supplementary Data
