# Supplementary material for: The chloride channel family gene CLCd negatively regulates pathogen-associated molecular pattern (PAMP)-triggered immunity in Arabidopsis
Source: J Exp Bot. 2014 Jan 21;65(4):1205–15. doi: 10.1093/jxb/ert484 (PMC3935575; doi:10.1093/jxb/ert484)
Supplement: Supplementary Data [file supp_ert484_jexbot107367_file001.pdf]

Supplementary Table S1. Sequences of the primers used in this work. Primers were used for semi-quantitative RT-PCR (RT), quantitative RT-PCR (qRT) and generating constructs ("Prom" for amplifying the native promoter from genomic DNA; "Cds" for amplifying CDS from cDNA)

Name	Sequence (5' to 3')
RT-ClCa-c1	GGTTTCACACATCGAGAGTTTAG
RT-ClCa-c2	AATGTAGTAGCCGACGGCGAGAA
RT-ClCb-c1	CCAACATGTTTGGTGCTACTAC
RT-ClCb-c2	CTGACAGCATCATCGTTGGTG
RT-ClCc-c1	CTTGGAAGTGGCTCAGATTCTTC
RT-ClCc-c2	CTCCAGCAAGAATGACAGGAATG
RT-CLCd-c1	TGGAATTGATATTCCCGGCACC
RT-CLCd-c2	ACCTAAAAGATCGTCTAGAAGC
RT-CLCd-c3	CTCCGATAACTCCTATTACAGC
RT-ClCe-c1	CCTTGATGATTCTCTCAACCAATC
RT-ClCe-c2	GATTGCTCAGCACCTTAAGTGAG
RT-ClCa-d1	GGAACACAACGCATAACGCATTC
RT-ClCa-d2	TCTAGCTTTTCCACTTTTGTG
RT-ClCb-d1	CTCCACGGGTTGATCTTGAGAG
RT-ClCb-d2	GTATGCCCTTAGGTCCTGTCTG
RT-ClCc-d1	CAAAGCCCGTGACTTTGGTAAAG
RT-ClCc-d2	CACTTGAGGGGATCAATGTGAG
RT-ClCd-d1	GCTTCTAGACGATCTTTTAGGTTAA
RT-ClCd-d2	ATGGCGGTATGACCGATAAATTC
RT-ClCe-d1	GCTGCACCAGCCAACTCATTC
RT-ClCe-d2	GCTCATCAGAGGAAAGGAGTGAG
RT-ClCf-F	GCGTTGATGATCGAAATGAC GAG
RT-ClCf-R	AGTCTCTGTAGACGAAGCCATGC
RT-ClCg-c1	CAAACTCAACGACGGAGGAC
RT-ClCg-c2	CTGACCAAGTATAGATGCAACAC
RT-ClCd-u1	CGAATCATCTCCAGAACGGGATC
RT-ClCd-u2	GACCAAGCAACGATGCAATGCAG
RT-FLS2 F	ACTCTCCTCCAGGGGCTAAG
RT-FLS2 R	GGGATGGTTCCAGTCAACAAG
RT-ACT7 F	GGTGAGGATATTCAGCCACTTGTCTG
RT-ACT7 R	TGTGAGATCCCGACCCGCAAGATC
qRT-ClCd F	CGGAGGTGTCAATAGTCTCG
qRT-ClCd R	GCAAATTTCCATCCAGCGAA
qRT-FRK1 F	ACGGGCATAGTTCCACAAAG
qRT-FRK1 R	CGTCAAAAGAACGACGATGA
qRT-PR1 F	GTTCTTCCCTCGAAAGCTCAAG
qRT-PR1 R	GTTACACCTCACTTTGGCACATC
qRT-AT1G51890 F	CTAGCCGACTTTGGGCTATC
qRT-AT1G51890 R	CCAGTTTGTTCTGTAATACTCAGG
qRT-AT2G17740 F	CATGCGTTGCTGAAGAAGAGG

qRT-AT2G17740 R	TGCTCCATCTCTCTTTGTGCC
qRT-FLS2 F	ACTCTCCTCCAGGGGCTAAG
qRT-FLS2 R	GGGATGGTTCCAGTCAACAAG
qRT-ACT2 F	AGTGTCTGGATCGGTGGTTC
qRT-ACT2 R	CCCCAGCTTTTTAAGCCTTT
Cds-FLS2 F	CGGGATCCATGGAAGTTGAGAGAGATCAACACATTTC
Cds-FLS2 R	CATGCCATGGTTGCAACACATTCTTGTACTTTCCATTG
Prom-ClCd F	CGGAATTCGACTTTGTGGCTGAGAGTGAG
Prom-ClCd R	CGGGATCCGATCGAGAGTTCGATCTCTGG
Cds-ClCd F	CGCGGATCCATGTTATCGAATCATCTCCAGAACG
Cds-ClCd R	GTTGCGGCCGCTTAACCTAAAAGATCGTCTAGAAGCG

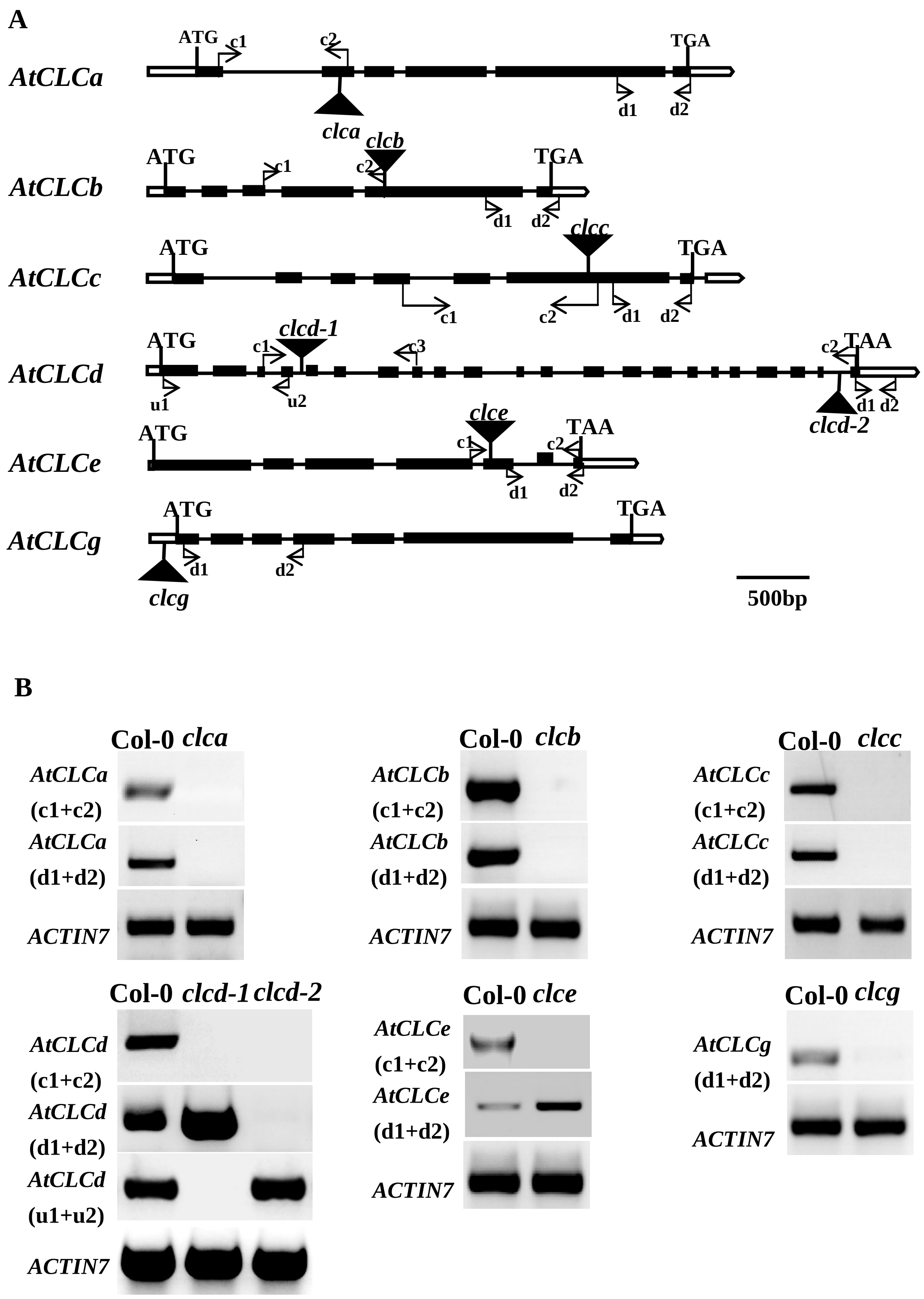

Supplementary Figure S1. Characterization of *Arabidopsis CLC* T-DNA insertion lines. A. Schematic structure of *AtCLC* family genes, T-DNA insertion sites (black arrows) and positions of the primers used in the semi-quantitative RT-PCR analysis. Filled boxes, exons; open regions, untranslated regions; black lines, introns. B. Expression of *AtCLC* family genes determined by semi-quantitative RT-PCR in *clc* mutant leaves. The level of *ACTIN7* transcripts was used as a loading control.

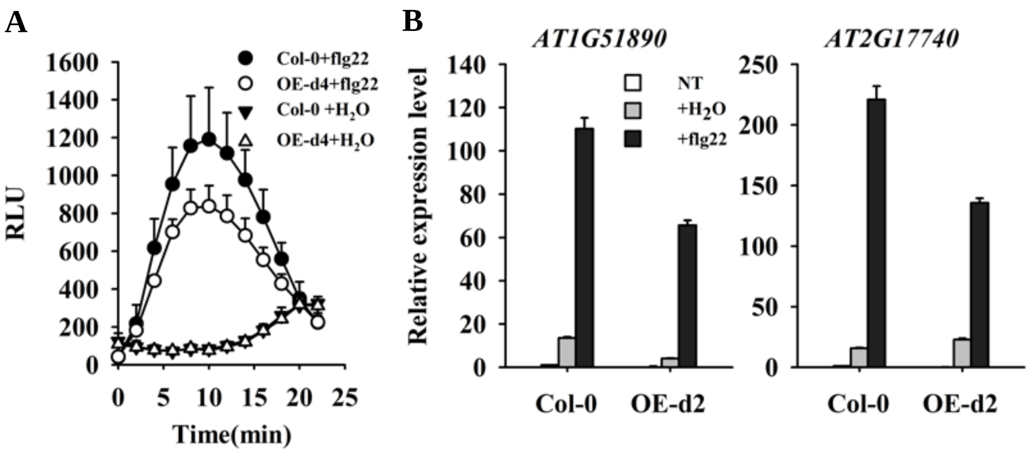

Supplementary Figure S2. PAMP-triggered immunity is compromised in the *AtCLCd* overexpressing lines. A. A reduced flg22-induced ROS burst was observed in an *AtCLCd*-overexpressing line (OE-d4). Values are mean ± SD (n=8) measured in RLU (Relative Light Units). B. Quantitative RT-PCR analysis of PTI marker gene expression after infiltration of 1μM flg22 or water for 1 hour. RNA was extracted from 4-week-old Col-0 and OE-d2 leaves. Samples were measured in triplicate and normalized to *ACTIN2*. Error bars indicate ± SD. NT, no treatment.

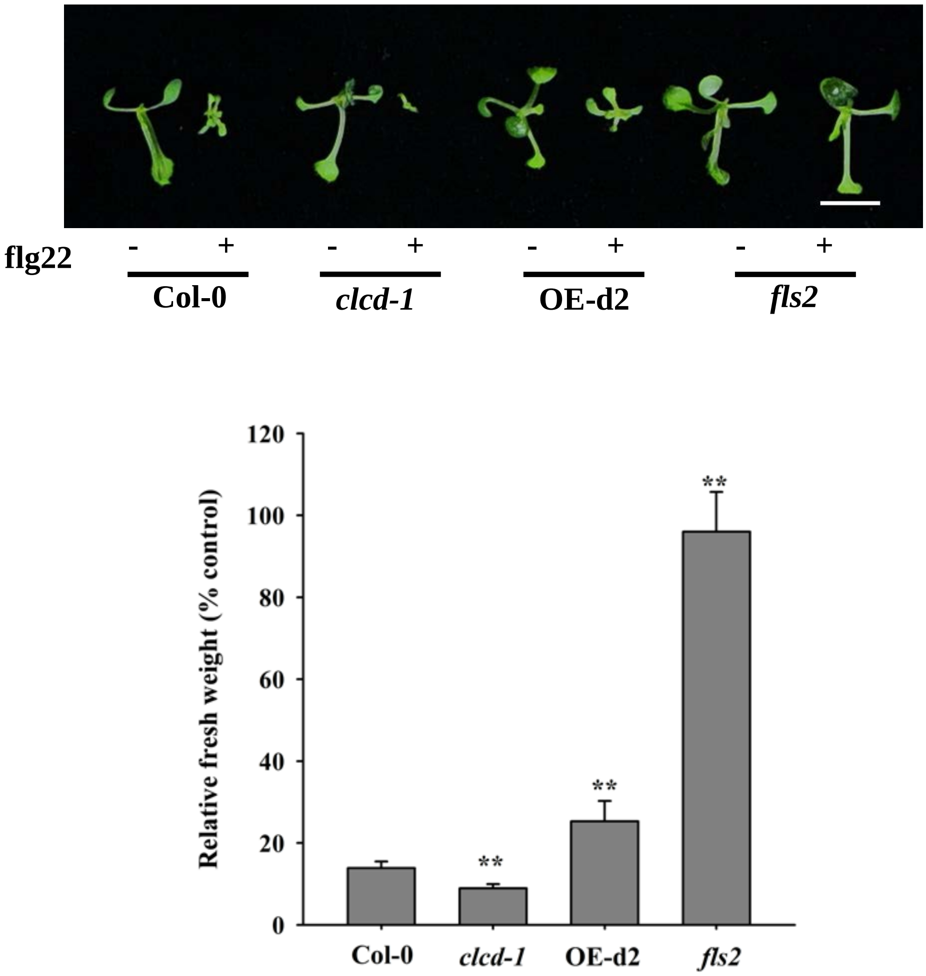

Supplementary Figure S3. Shoot growth inhibition induced by flg22 in Col-0, *clcd* mutant and *AtCLCd*-overexpressing plants. Five-day-old seedlings were treated or mock treated with 1μM flg22 for eight days. Representative pictures were taken and seedling fresh weights were measured. Results are means±SD (n=11-14). ** P<0.01 (*t*-test). Bar =1cm.

A

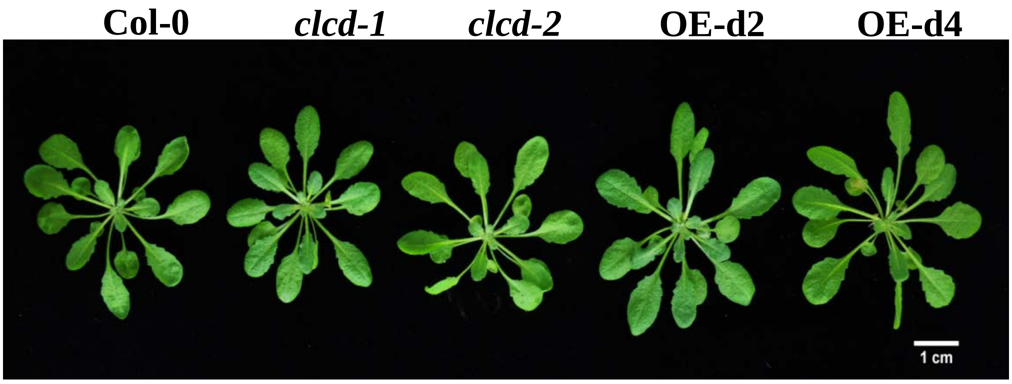

B

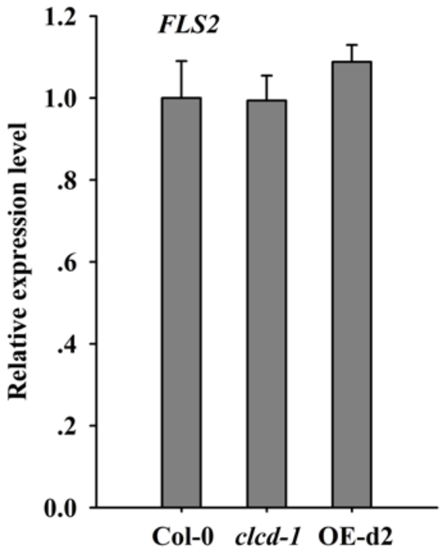

Supplementary Figure S4. Morphological phenotypes and *FLS2* expression levels in the *AtCLCd*-misexpressing plants. A. 5-week-old plants grown in soil under short-day conditions are shown. Bar =1cm. B. Expression levels of *AtCLCd* were determined by quantitative RT-PCR in the wild-type (Col-0), the *clcd* mutant and the *AtCLCd*-overexpressing lines. Samples were measured in triplicate and the data were normalized to *ACTIN2*. Error bars indicate SD.
